# MYO19 is associated with tumor progression, immune evasion, ferroptosis-related signatures in lung squamous cell carcinoma

**DOI:** 10.3389/fonc.2025.1727301

**Published:** 2026-01-06

**Authors:** Fang Zhao, Guiying Chen, Jianfeng Pan, Yongxiang Zhang, Wei Li, Kaifeng Niu, Hui Ma

**Affiliations:** 1 Department of Respiratory and Critical Care Medicine, Tianjin Chest Hospital, Tianjin University, Tianjin, China; 2 China National Center for Bioinformation, Beijing, China; 3 Beijing Institute of Genomics, Chinese Academy of Sciences, Beijing, China

**Keywords:** ferroptosis, hsa-miR-520a-3p, immunosuppression, lung squamous cell carcinoma, MYO19, tumor microenvironment

## Abstract

**Background:**

Lung squamous cell carcinoma (LUSC) is a highly aggressive malignancy with limited targeted therapies and poor clinical outcomes. Ferroptosis, an iron-dependent form of regulated cell death, plays a crucial role in tumor progression, metabolic reprogramming, and immune modulation. Increasing evidence suggests that dysregulation of ferroptosis contributes to therapeutic resistance and immune escape in various cancers. MYO19, a mitochondrial trafficking protein, has recently been implicated in oxidative stress and metabolic control, but its role in ferroptosis and tumor immunity remains unclear. Meanwhile, microRNAs (miRNAs) are recognized as key post-transcriptional regulators in cancer biology. Among them, hsa-miR-520a-3p has been reported to exhibit tumor-suppressive functions in several malignancies. However, the interplay between hsa-miR-520a-3p and MYO19, and their potential involvement in ferroptosis regulation and immune modulation in LUSC, has not been systematically investigated.

**Methods:**

Data were collected from TCGA, UCSC XENA, ENCORI, HPA, and UALCAN public database. Differential expression, prognostic, correlation analyses and miRNA analyses were performed using bioinformatics tools including TIMER, TISIDB, Kaplan-Meier Plotter, and ENCORI. Ferroptosis-related analysis utilized Ze-Xian Liu’s dataset. Functional assays, including CCK-8 viability, Transwell migration, and MDA/GSH measurements, were performed in NCI-H226 and NCI-H2170 cells after transfection with miR-520a-3p mimics/inhibitors or MYO19 knockdown/overexpression constructs. Ferroptosis sensitivity was further tested under RSL3 treatment, and ferroptosis protein markers as well as rescue experiments were analyzed by Western blotting.

**Results:**

The result revealed that MYO19 was significantly upregulated in multiple tumor types and correlated with unfavorable prognosis. Especially in LUSC, elevated MYO19 expression was associated with advanced stage, reduced immune infiltration, and enrichment of ferroptosis-resistant transcriptional programs, whereas hsa-miR-520a-3p showed opposite patterns. Overexpression of hsa-miR-520a-3p in NCI-H226 and NCI-H2170 cells increased lipid peroxidation (MDA increased), reduced intracellular GSH, and enhanced RSL3-induced cytotoxicity, indicative of ferroptosis activation. Conversely, MYO19 knockdown elevated ACSL4 and reduced SLC7A11, changes that were partially reversed by MYO19 re-expression. These findings suggest that the hsa-miR-520a-3p/MYO19 axis is associated with ferroptosis susceptibility and may influence the immunosuppressive tumor microenvironment.

## 1 Introduction

Lung squamous cell carcinoma (LUSC), a major subtype of non-small cell lung cancer (NSCLC), accounts for approximately 20–30% of all lung cancer cases worldwide. Despite advances in targeted therapy and immunotherapy, the five-year survival rate for patients with advanced-stage LUSC remains below 20%, largely due to late-stage diagnosis, aggressive tumor progression, and resistance to current treatments (1–4). This underscores the urgent need to uncover novel molecular mechanisms and identify new therapeutic targets to improve patient outcomes.

The interaction between LUSC and the immune system is mediated by complex regulatory networks within the tumor microenvironment (TME). Recent studies have revealed stage-dependent immunological features in LUSC: early-stage tumors exhibit enhanced cytotoxic activity and increased neoantigen load, while advanced stages are characterized by immunosuppressive infiltration, particularly of M0/M2 macrophages and plasma cells (5). Furthermore, immune cell dynamics—such as changes in dendritic cells, eosinophils, mast cells, and memory CD4^+^ T cells—have been closely linked to disease progression and prognosis. Smoking, a primary risk factor, exacerbates immune dysregulation by reprogramming tumor metabolism, impairing natural killer (NK) cell function, and fostering an immunosuppressive microenvironment through endothelial dysfunction and mast cell dormancy (6).

MicroRNAs (miRNAs) are emerging as key regulators in cancer biology, influencing tumor growth, immune evasion, and ferroptosis—a form of regulated cell death driven by iron-dependent lipid peroxidation (7, 8). For example, miR-665 promotes proliferation via the Wnt5a/β-catenin pathway and suppresses apoptosis by targeting Caspase-3, while LINC01607 acts as a competing endogenous RNA (ceRNA) to enhance mitophagy and drug resistance by sequestering miR-892b and increasing P62 expression (9, 10). Notably, hsa-miR-520a-3p has been identified as a tumor suppressor in acute myeloid leukemia and esophageal squamous cell carcinoma, but its role in LUSC remains undefined (11, 12).

MYO19, a mitochondrial actin-based motor protein, has been implicated in mitochondrial trafficking and cancer progression (13, 14). Its deficiency enhances reactive oxygen species (ROS) gradients, potentially promoting metastasis (15). ROS are key mediators in cancer biology, modulating oncogenic signaling, immune responses, and genomic instability (16). Ferroptosis intersects with ROS signaling and mitochondrial metabolism, positioning it as a promising target for overcoming therapy resistance and immune evasion in cancer (17–19).

In this study, we investigate the role of MYO19 in LUSC, with a particular focus on its regulation by hsa-miR-520a-3p and its impact on ferroptosis. Our findings reveal that MYO19 overexpression is associated with features of tumor progression and ferroptosis resistance in LUSC and is negatively regulated by hsa-miR-520a-3p. This regulatory axis may contribute to immune evasion and ferroptosis resistance, offering novel insights into the pathogenesis of LUSC and potential therapeutic strategies. However, further mechanistic studies are required to establish causality.

## 2 Methods

### 2.1 Data collection

We retrieved data containing mRNA, miRNA, and related clinical information from the Cancer Genome Atlas (TCGA, https://portal.gdc.cancer.gov/repository), UCSC XENA (https://xenabrowser.net), ENCORI (https://rnasysu.com/encori/panCancer.php), and the Human Protein Atlas portal (HPA, https://www.proteinatlas.org/).

### 2.2 Differential expression analysis

We downloaded uniformly standardized cancer datasets (TCGA TARGET GTEx, PANCAN, N = 19131, G = 60499) from the UCSC database (https://xenabrowser.net/) and extracted MYO19 gene expression data across samples. We selected samples with sources such as Solid Tissue Normal and Primary Solid Tumor, applied log2(x + 0.001) transformation to each expression value, and excluded cancer types with fewer than three samples. We also obtained MYO19 protein expression data from the UALCAN portal using CPTAC data and assessed MYO19’s diagnostic value using TCGA RNA sequencing data.

### 2.3 Prognostic analysis

We downloaded LUSC STAR-counts data and corresponding clinical information from the TCGA database (https://portal.gdc.cancer.gov), extracted TPM formatted data, and normalized it using log2(TPM + 1). We retained samples with both RNAseq data and clinical information for subsequent analysis. We performed univariate and multivariate Cox proportional hazards regression analyses, visualized results with forest plots, and constructed a Nomogram to predict 5-year overall survival. We used R software (v4.0.3) for statistical analysis, considering results significant when P < 0.05. Additionally, we used the Kaplan-Meier Plotter (http://kmplot.com/analysis/) to study the correlation between MYO19 expression and OS across different tumor types.

### 2.4 Correlation between MYO19 and immunity

We used TIMER (https://cistrome.shinyapps.io/timer/) to analyze the correlation between MYO19 expression levels and immune cell infiltration or immune checkpoint expression in multi-cancer tissues. We used TISIDB to analyze the relationship between MYO19 and chemokines, chemokine receptors, and MHC molecules, and explored the correlation between MYO19 expression and stromal scores, immune scores, estimate scores, and TIICs across various cancers using the SangerBox website. Spearman’s rank correlation analysis was applied to evaluate the relationship between variables. All correlation analyses were conducted using the cor.test function in R (version 4.2.2), with two‐tailed tests and a significance threshold of p < 0.05.

### 2.5 Correlation between MYO19 and Related miRNAs

We studied MYO19-related miRNA regulatory networks in LUSC. We predicted potential upstream miRNAs using tools like starbase (https://rnasysu.com/encori/panMirCoExp.php#) and miRDB (https://mirdb.org/mirdb/index.html), selecting only intersecting miRNAs as candidates. Based on the ceRNA hypothesis, we assessed the correlation between candidate miRNAs and MYO19 expression using the ENCORI platform, which uses TCGA project expression data and miRNA-seq data. In this study, the expression of miR-520a-3p was analyzed using publicly available TCGA datasets for LUSC. Experimental validation in patient samples was not performed, and the findings should not be considered as clinical diagnostic claims.

### 2.6 Correlation with ferroptosis-related genes

Ferroptosis-related genes were sourced from Ze-Xian Liu’s systematic analysis of ferroptosis in cancers (20). We used the Wilcoxon test to assess significance between sample groups, considering results significant when P < 0.05.

### 2.7 Cell culture and antibodies

HEK293, NCI-H226 and NCI-2170 cells were obtained from the Cell Bank of the Chinese Academy of Sciences (Shanghai, China) and were routinely tested negative for mycoplasma before experiments. HEK293 cells were maintained in Dulbecco’s modified Eagle’s medium (DMEM). NCI-H226 and NCI-2170 cells were maintained in RPMI-1640 medium. All media were supplemented with 10% fetal bovine serum (FBS; Gibco) and 1% penicillin–streptomycin (100 U/mL penicillin, 100 μg/mL streptomycin). Cells were cultured at 37 °C in a humidified incubator with 5% CO_2_. The following primary antibodies were used for immunoblotting: MYO19 (ab116792; Abcam), ACSL4 (PAB34846, Bioswamp), SLC7A11 (A2413, Abclonal) and GAPDH (MAB374; Millipore).

### 2.8 Glutathione assay and MDA assay

Total Glutathione Assay Kit (S0052; Beyotime) and MDA Assay Kit (S0131M; Beyotime) were used to quantify intracellular GSH and MDA according to the manufacturer’s protocols. Briefly, NCI-H226 cells were first transfected with either a negative control or miR-520a-3p for 12 h, followed by transfection with Flag-EV or Flag-MYO19 plasmids for an additional 24 h. The cells were then treated with 20 μM RSL3 (SML2234; Sigma-Aldrich) for 24 h. Cells were then harvested and lysed, and GSH and MDA levels were measured per kit instructions and normalized to total protein content where applicable. Results from at least three independent experiments are reported.

### 2.9 CCK-8 assay

For cell viability measurements, 2,000 NCI-H226 cells per well were seeded into 96-well plates and transfected with negative control or miR-520a-3p inhibitor/mimic as indicated. At 1, 2, 3, and 4 days after seeding, 10 μL of CCK-8 reagent (C0042; Beyotime) was added to each well and incubated for 1 h at 37 °C in the dark. Absorbance at 450 nm was read on a microplate reader (Bio-Rad, USA) using cell-free wells as blanks. Experiments were performed in triplicate.

### 2.10 Transwell assay

For Transwell migration, 5,000 NCI-H226 cells transfected with negative control or miR-520a-3p inhibitor were resuspended in serum-free medium and seeded into the upper chambers of 24-well Transwell inserts (ECM508; Millipore Sigma). The lower chambers contained medium supplemented with 10% FBS as a chemoattractant. After 48 h at 37°C, non-migrated cells on the upper surface were gently removed. Migrated cells on the lower membrane were stained with the kit’s cell stain reagent for 20 min, imaged using a Leica DM5000 microscope, and quantified by counting five random fields per insert.

### 2.11 Real-time quantitative polymerase chain reaction

Trizol reagent (Invitrogen) was used to extracted total RNA according to the producer’s instruction. For miR‐520a‐3p detection, cDNA was synthesized by using the miRNA 1st strand cDNA Synthesis Kit (Vazyme, MR101,China). For the detection of MYO19, total RNA was reversely transcribed into complementary DNA (cDNA) using RevertAid First Strand cDNA Synthesis Kit (K1622, Thermo Fisher Scientific) and quantitative PCR was then carried out with TB Green™ Premix Ex Taq™ (RR420A, TaKaRa). The level of mRNA was compared with GAPDH, and the level of miRNA was compared with U6. The primers applied for qPCR analysis were synthesized by Tsingke Biotechnology Co., Ltd. (China) in this study and were as follows:

miR‐520a‐3p: F, 5′‐ACACTCCAGCTGGGAAAGTGCTTCCC‐3′, R, 5′‐CTCAACTGGTGTCGT GGA‐3′. U6: F 5′‐CTCGCTTCGGCAGCACA‐3′, R, 5′‐ACGCTTCACGAATTTGCGT‐3′. MYO19:F, 5′‐AGCCCATATCCTGCCAAAG‐3′, R, 5′‐CAGCCGAGTCACCAGTTATG‐3′.

GADPH: F 5′‐GATATTGTTGCCATCAATGAC‐3′, R 5′‐TTGATTTTGGAGGGATCTCG‐3′;

The miRNA inhibitor and mimics for hsa-mir-520a-3p used in this study were generated by GenePharma (Shanghai, China) and transfected into NCI-H226 or NCI-H2170 cells using Lipofectamine 2000 (11668019, Thermo Fisher Scientific) according to the manufacturer’s protocol. The sequences targeting to hsa-mir-520a-3p were as follows: hsa-mir-520a-3p inhibitor (B03001, GenePharma, China): 5′‐ACAGUCCAAAGGGAAGCACUUU‐3′. hsa-mir-520a-3p mimics (B02001, GenePharma, China): F, 5′‐AAAGUGCUUCCCUUUGGACUGU‐3′, R, 5′‐AGUCCAAAGGGAAGCACUUUUU‐3′.

### 2.12 Luciferase reporter assay

Candidate miRNAs upstream of MYO19 and their predicted binding sites were obtained from public databases. Wild-type and mutant MYO19 3′UTR reporter plasmids were co-transfected with the indicated miRNA mimics into HEK293 cells using LipoMax (LipoMax32012; Sudgen) following the manufacturer’s protocol. At 48 h post-transfection, luciferase activities were measured with the Dual-Glo^®^ Luciferase Assay System (E2920; Promega); firefly signals were normalized to Renilla for each well. Data represent at least three independent experiments.

### 2.13 Statistical analyses

The statistical analyses in this study were performed using the aforementioned online databases. Experimental data are presented as the mean ± SD of three independent biological experiments. R (v4.0.3) was used for statistical analysis, with the Wilcoxon test for two-sample comparisons and the Kruskal-Wallis test for three or more samples. P-value < 0.05 was considered statistically significant.

## 3 Result

### 3.1 Cross-cancer profiling and LUSC-specific overexpression of MYO19

**Figure 1:**
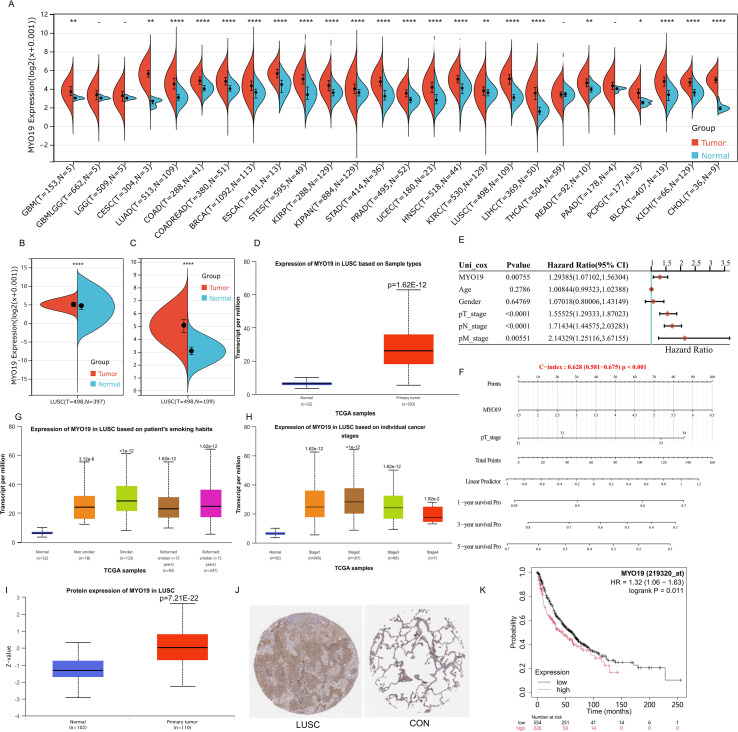
MYO19 expression in cross-cancer and its prognostic significance in LUSC. **(A)** MYO19 expression across various cancer types and corresponding normal tissues in the TCGA and GTEx datasets. **(B, C)** Violin plots depicting the differential expression of MYO19 in LUSC tumor tissues compared to normal tissues in the TCGA and GTEx datasets (p < 0.0001). **(D)** Scatter and bar plot showing MYO19 expression levels in individual LUSC samples and the expression levels in tumor and normal groups. **(E)** Forest plot of univariate Cox regression analysis showing the prognostic significance of MYO19 expression and other clinical variables (e.g., pT_stage, pN_stage, and pM_stage) in LUSC. **(F)** Nomogram integrating MYO19 expression and clinical features to predict 1-, 3-, and 5-year survival probabilities for LUSC patients. **(G)** Boxplot showing the relationship between MYO19 expression and smoking status. **(H)** MYO19 expression across different LUSC stages. **(I)** Boxplot depicting MYO19 protein expression levels in LUSC tumors versus normal tissues (p < 0.001). **(J)** Representative immunohistochemical staining of MYO19 in LUSC tumor and normal tissues. Tumor tissues show strong MYO19 staining, while normal tissues exhibit weak or no staining. **(K)** Kaplan-Meier survival curve showing overall survival of LUSC patients stratified by MYO19 expression. *p<0.05, **p<0.01,****p<0.0001.

A cross-cancer comparative analysis based on TCGA data revealed significant upregulation of MYO19 in multiple cancer types. Transcriptional profiling of 33 cancer types demonstrated significant MYO19 elevation in 68.2% of malignancies compared to matched normal tissues, with LUSC showing the pronounced differential expression (**Figure 1A**). These findings establish MYO19 as a multi-cancer oncogenic signature, particularly in lung squamous carcinomas.

LUSC-specific validation confirmed this pattern through multi-omics integration. **Figures 1B** and **1C** depict this upregulation of MYO19 mRNA expression in both the TCGA and GTEx datasets, with a consistent and robust statistical significance (p < 0.0001). Consistently, MYO19 mRNA levels were markedly higher in LUSC tissues than in normal lung tissues, as evidenced by data from the CPTAC database (**Figure 1D**).Overall, the data demonstrate a pronounced overexpression of MYO19 in LUSC, indicating its potential role in tumor biology.

### 3.2 Prognostic significance of MYO19 in LUSC

Next, we investigated whether MYO19 serves as an independent prognostic factor in LUSC. Results from a univariate Cox regression analysis (**Figure 1E**) reveal that elevated MYO19 expression is significantly associated with poorer overall survival (HR = 1.298, 95% confidence interval CI: 1.070–1.353, p = 0.00075). Other clinical variables, including pT_stage, pN_stage, and pM_stage, also exhibit significant associations with patient outcomes. A nomogram analysis (**Figure 1F**, **Supplementary Figure S2**, **Supplementary Table S1**) further confirms these findings by integrating MYO19 expression and key clinical features to predict 1-, 3-, and 5-year survival probabilities. In addition, univariate cox analysis across different stages (N0, N1, and N2) showed that MYO19 expression is associated with poor prognosis in stage N0 (HR = 1.4, p = 0.00141). These suggest the potential prognostic significance of MYO19 in LUSC.

The link between MYO19 expression and patient prognosis is reinforced by Kaplan-Meier survival analysis (**Figure 1K**). Patients with high MYO19 expression have significantly worse overall survival than those with low expression (log-rank p = 0.011, HR = 1.32, 95% CI: 1.10–1.56). These results strongly suggest that MYO19 serves as a reliable marker of poor prognosis in LUSC. Beyond its prognostic value, MYO19 expression is closely associated with clinical and behavioral factors. For instance, MYO19 expression is significantly higher in former and current smokers than in non-smokers, as shown in **Figure 1G**. This finding points to a potential connection between smoking and MYO19 upregulation in LUSC.

Moreover, we examined the expression levels of MYO19 mRNA and protein across different cancer stages. **Figure 1H** illustrates a progressive increase in MYO19 mRNA expression with advancing cancer stage, indicating its potential role in tumor progression and aggressiveness. At the protein level, MYO19 expression is markedly higher in LUSC tumor tissues than in normal tissues (p < 0.001, **Figure 1I**). Immunohistochemical analysis (**Figure 1J**) corroborates this observation, showing strong MYO19 staining in LUSC tissues and weak or absent staining in normal tissues. Collectively, these findings highlight the elevated expression of MYO19 in LUSC and suggest its potential role in driving disease progression and unfavorable clinical outcomes.

### 3.3 Correlation between MYO19 expression and immune-related genes in LUSC

**Figure 2:**
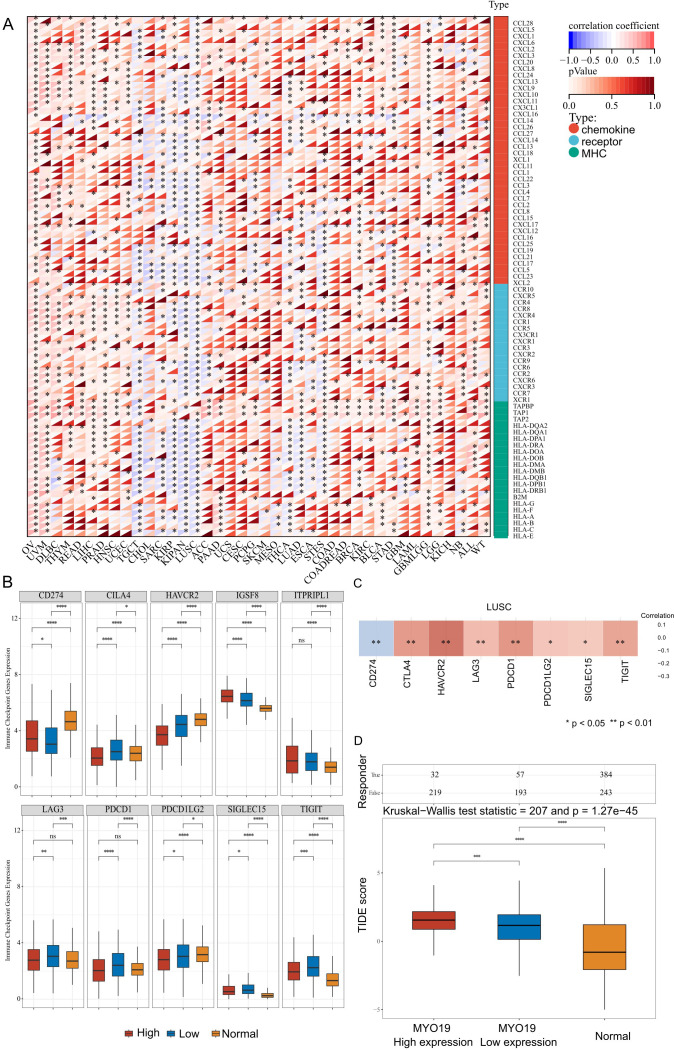
Correlation of MYO19 expression with immune-related genes and immune microenvironment in LUSC. **(A)** Heatmap showing the correlation between MYO19 expression and immune-related gene families, including chemokines, chemokine receptors, and MHC genes across cancers. **(B)** Boxplots comparing immune checkpoint gene expression levels in LUSC patients with high and low MYO19 expression and in normal group (TCGA, n=49; GTEx, N = 578). **(C)** Heatmap illustrating the correlation between MYO19 expression and immune checkpoint genes in LUSC (TCGA+GTEx, N = 498). **(D)** Boxplots showing the TIDE scores for MYO19-high, MYO19-low, and normal group (TCGA, n=49; GTEx, N = 578) in LUSC. *p<0.05, **p<0.01,***p<0.001, ****p<0.0001.

To investigate the divergent correlations between MYO19 expression and immune-related gene families across various cancer types, we analyzed the correlation matrix of MYO19 expression with chemokines, chemokine receptors, and MHC genes in various cancers. As shown in **Figure 2A**, MYO19 shows widespread positive correlations with immune-related genes across most cancer types, its expression in LUSC exhibits a predominantly negative correlation with these genes. This unique pattern in LUSC suggests that MYO19 may disrupt immune-related pathways or immune gene expression, leading to a distinct tumor immune microenvironment in this cancer type. Further analysis of MYO19’s relationship with immune checkpoint genes provides additional insights. The expression levels of immune checkpoint genes are compared among LUSC patients with high and low MYO19 expression and normal subjects. CD274 (PD-L1) and IGSF8 are significantly upregulated in the MYO19-high group compared to the MYO19-low group, while most other immune checkpoint genes, including CTLA4, HAVCR2, LAG3, PDCD1 (PD-1), and TIGIT, are significantly downregulated in the MYO19-high group (**Supplementary Figure S2**). Notably, the expression levels of these immune checkpoint genes differ significantly between the LUSC groups and the normal group (**Figure 2B**). This inverse expression pattern suggests that MYO19 may selectively associated with immune checkpoint pathways, potentially diminishing the expression of certain immune checkpoints while upregulating others, such as PD-L1.

This complex relationship between MYO19 and immune checkpoint genes in LUSC is further explored in **Figure 2C**. Consistent with the overall pattern, CD274 (PD-L1) exhibits a positive correlation with MYO19 expression, while the majority of other immune checkpoint genes, including CTLA4, LAG3, and PDCD1, show negative correlations. These findings indicate that MYO19 expression is associated with PD-L1-related immune checkpoint pathways and higher TIDE scores, which are compatible with an immune-evasive phenotype.

Additionally, MYO19 expression is associated with an elevated TIDE score in LUSC, as shown in **Figure 2D**. The TIDE score, which evaluates tumor immune dysfunction and exclusion, is significantly higher in the MYO19-high group compared to the MYO19-low and normal groups (Kruskal-Wallis test, p < 0.001). A higher TIDE score suggests enhanced immune escape potential and a reduced likelihood of response to immune checkpoint inhibitors (e.g., PD-1/PD-L1 and CTLA-4 inhibitors). This implies that tumors with high MYO19 expression are more immunosuppressive and likely to evade immune surveillance, further limiting the efficacy of immunotherapy.

### 3.4 MYO19 expression is associated with tumor microenvironment features and proliferation signatures in LUSC

**Figure 3:**
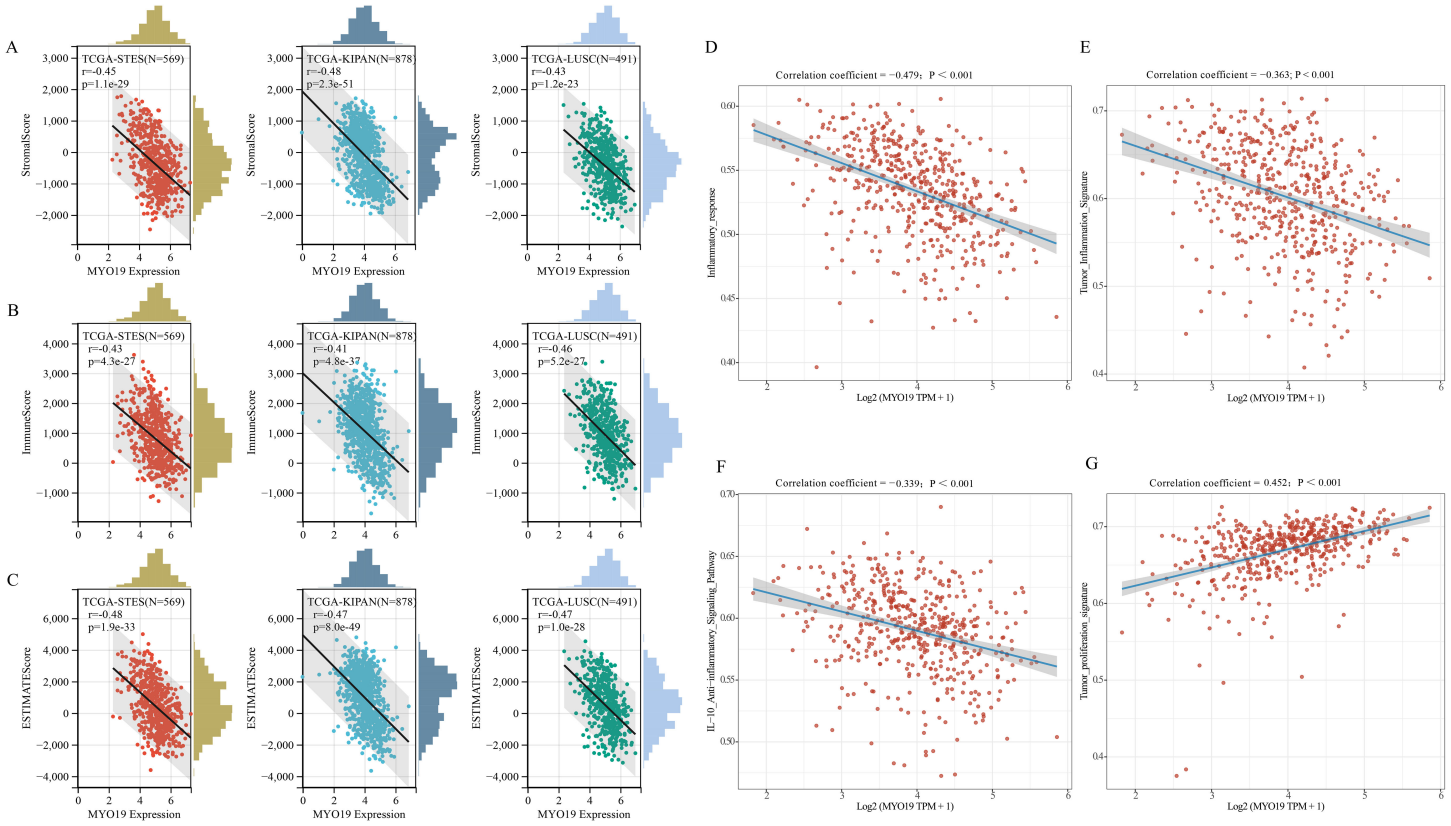
Correlation of MYO19 expression with tumor microenvironment scores, inflammation signatures, and proliferation in LUSC. **(A–C)** Scatter plots with histograms showing the correlation between MYO19 expression and microenvironment scores in LUSC, including the StromalScore **(A)**, ImmuneScore **(B)**, and ESTIMATEScore **(C)**. **(D, E)** Scatter plots showing correlations between MYO19 expression and inflammatory signatures in LUSC. **(F, G)** Scatter plots showing the correlation between MYO19 expression and tumor proliferation signatures in LUSC.

To further explore the role of MYO19 in the tumor microenvironment of LUSC, we assessed its correlation with microenvironmental scores. As shown in **Figures 3A–C**, MYO19 expression exhibits a negative correlation with the StromalScore. (r = -0.43, p = 1.2e-23), suggesting lower stromal content in MYO19-high tumors. Similarly, a strong negative correlation with the ImmuneScore (r = -0.46, p = 5.2e-27) indicates reduced immune cell infiltration, while the ESTIMATEScore, which combines immune and stromal cell abundance, also shows a significant inverse correlation (r = -0.47, p = 1.0e-28). Collectively, these findings suggest that tumors with high MYO19 expression are characterized by a poorly infiltrated and immunosuppressive microenvironment.

Beyond its impact on immune and stromal scores, MYO19 expression is inversely correlated with inflammatory activity in LUSC. As shown in **Figure 3D**, MYO19 expression negatively correlates with the inflammatory response signature (r = -0.479, p < 0.0001), suggesting suppression of inflammatory processes within the tumor microenvironment. Furthermore, MYO19 is negatively associated with the tumor inflammation signature (r = -0.363, p = 2.72e-17, **Figure 3E**), and IL-10 Anti-inflammatory Signaling Pathway (r = -3.39e-01;p =8.19e-15, **Figure 3F**) indicating that MYO19-high tumors have a reduced capacity to activate immune-stimulating inflammatory processes. These results imply that high MYO19 expression may facilitate immune evasion by suppressing immune-activating inflammatory responses.

In contrast to its negative associations with immune and inflammatory features, MYO19 expression shows a strong positive correlation with tumor proliferation. As illustrated in **Figure 3G**, MYO19 expression is positively associated with the tumor proliferation signature (r = 0.452, p < 0.0001), suggesting that MYO19 may promote tumor growth and cellular proliferation.

### 3.5 Identification and functional validation of the hsa-miR-520a-MYO19 axis in LUSC

**Figure 4:**
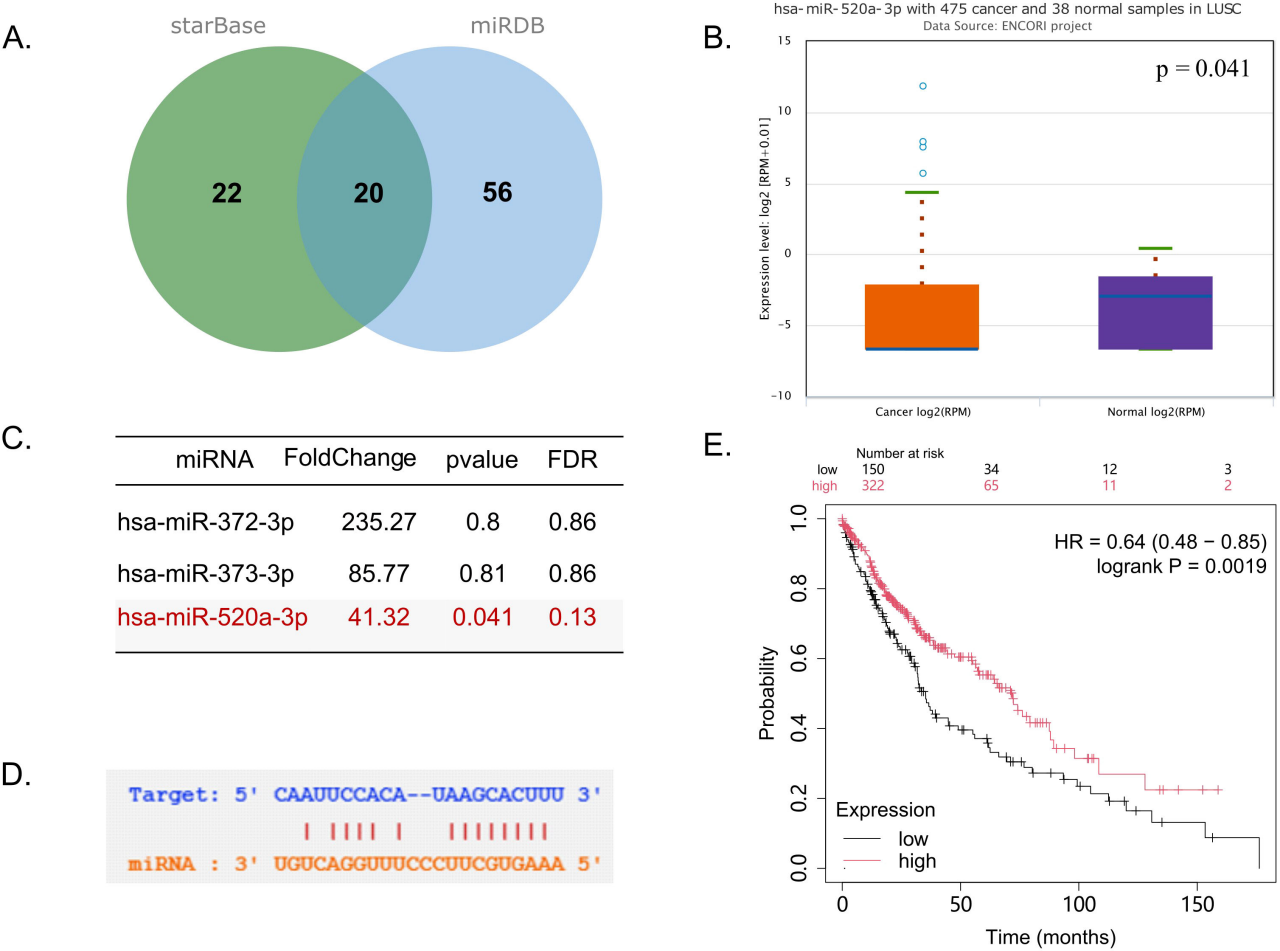
Identification and characterization of the hsa-miR-520a-3p-MYO19 regulatory axis in LUSC. **(A)** Venn diagram showing the overlap of predicted miRNAs targeting MYO19 from the starBase and miRDB databases. **(B)** Boxplot showing the expression of hsa-miR-520a-3p in LUSC tumor tissues compared to normal tissues. **(C)** Table summarizing the expression changes of three significantly negatively correlated miRNAs in lung squamous cell carcinoma. **(D)** Predicted binding site of hsa-miR-520a-3p in the 3’-UTR of MYO19 mRNA. **(E)** Kaplan-Meier survival curve for LUSC patients stratified by hsa-miR-520a-3p expression.

To explore the regulatory mechanism of MYO19 expression in LUSC progression, we conducted a comprehensive analysis using the starBase and miRDB databases. Among 20 miRNAs predicted to target MYO19, hsa-miR-520a-3p was identified as the strongest candidate, showing a significant correlation with MYO19 expression (**Figures 4A, C**). Bioinformatics analysis confirmed a direct binding site for hsa-miR-520a-3p in the 3’-UTR of MYO19, supporting its regulatory role (**Figure 4D**). Expression analysis revealed that hsa-miR-520a-3p was markedly downregulated in LUSC tumors compared to adjacent normal tissues (fold change = 41.32, p = 0.041; **Figures 4B, C**, **Supplementary Figure S3**), consistent with its tumor-suppressive role. Kaplan-Meier survival analysis demonstrated that patients with high hsa-miR-520a-3p expression had significantly better overall survival compared to those with low expression (HR = 0.64, 95% CI: 0.48–0.85, log-rank p = 0.0019; **Figure 4E**). These findings collectively suggest a potential link between the hsa-miR-520a-3p-MYO19 axis as a prognostic marker and therapeutic target in LUSC.

**Figure 5:**
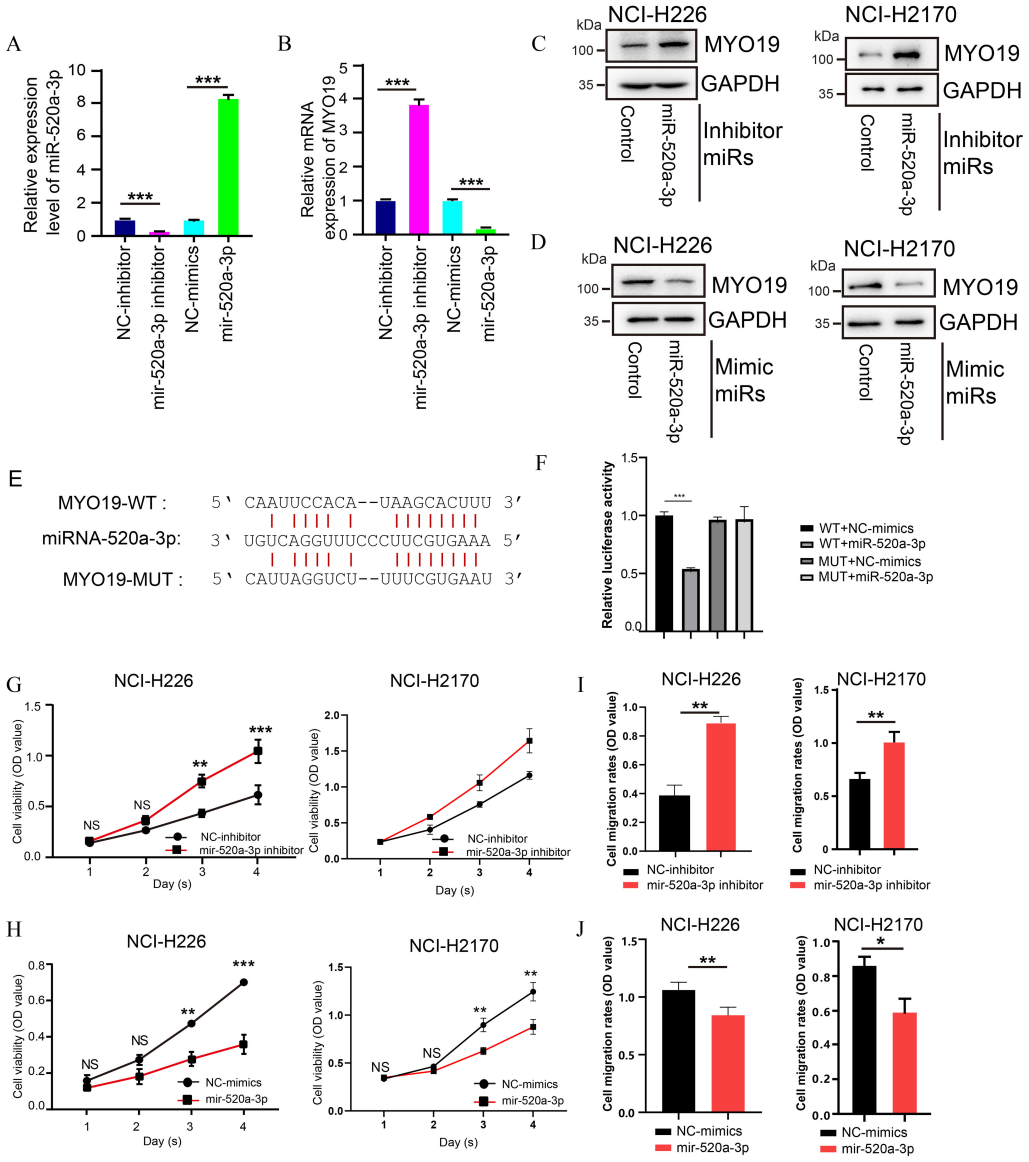
Functional validation of the hsa-miR-520a-3p-MYO19 axis in LUSC cell lines. **(A, B)** Relative expression levels of hsa-miR-520a-3p and MYO19 mRNA after transfection with hsa-miR-520a-3p inhibitors and mimics. **(C, D)** Western blot analysis of MYO19 protein levels in NCI-H226 and NCI-H2170 cells after transfection with hsa-miR-520a-3p inhibitors or mimics. **(E)** Predicted binding sites of hsa-miR-520a-3p in the 3’UTR of MYO19; **(F)** The wild-type or mutated binding sites of MYO19 were cloned separately into the pGL3-control vector. The above plasmids and pRL-TK plasmids were transfected into HEK293 cells with hsa-miR-520a-3p mimics and luciferase activities were measured. **(G, H)** Cell viability assays showing the effect of hsa-miR-520a-3p inhibitors **(G)** and mimics **(H)** on NCI-H226 and NCI-H2170 cell proliferation. **(I, J)** Transwell migration assays demonstrating the impact of hsa-miR-520a-3p inhibitors **(I)** and mimics **(J)** on NCI-H226 cell migration. *p<0.05, **p<0.01, ***p<0.001.

To validate this regulatory axis at the experimental level, miRNA mimic and inhibitor experiments were performed in LUSC cell lines (NCI-H226 and NCI-H2170). The expression of hsa-miR-520a-3p was significantly reduced upon inhibitor treatment and markedly increased with mimic treatment (p < 0.001; **Figures 5A, B**). Western blot analysis confirmed that MYO19 protein expression was upregulated upon inhibition of hsa-miR-520a-3p and downregulated following its overexpression in NCI-H226 and NCI-H2170 cells (**Figures 5C, D**). To further confirm whether MYO19 is directly targeted by hsa-miR-520a-3p, wild-type (WT) and mutant MYO19 luciferase reporter plasmids were constructed and co-transfected into HEK293 cells with hsa-miR-520a-3p mimics for 48 h. The dual-luciferase assay results revealed that hsa-miR-520a-3p mimics markedly decreased the luciferase activity of MYO19-WT, but not MYO19 mutant (MUT) (**Figures 5E, F**). These results indicated that hsa-miR-520a-3p negatively regulates MYO19 via targeting at the 3’UTR.

Functional assays further revealed the tumor-suppressive role of hsa-miR-520a-3p in LUSC cell lines. Cell viability assays of NCI-H226 and NCI-H2170 cells showed that hsa-miR-520a-3p inhibition significantly increased cell proliferation, while overexpression of hsa-miR-520a-3p suppressed cell growth over four days (p < 0.01; **Figures 5G, H**). Similarly, transwell migration assays of NCI-H226 and NCI-H2170 cells demonstrated that hsa-miR-520a-3p inhibition significantly enhanced cell migration, whereas hsa-miR-520a-3p overexpression suppressed migration (p < 0.01; **Figures 5I, J**). These findings indicate that hsa-miR-520a-3p may act as a tumor suppressor by inhibiting both proliferation and migration of LUSC cells.

These results establish the hsa-miR-520a/MYO19 axis as a critical pathway in LUSC progression. The downregulation of hsa-miR-520a-3p in tumors disrupts its regulatory suppression of MYO19, leading to MYO19 overexpression and potentially contributing to immune evasion, tumor proliferation, and migration. This regulatory axis not only provides mechanistic insights into LUSC progression but also highlights hsa-miR-520a-3p as a potential therapeutic target and prognostic biomarker for improving patient outcomes.

### 3.6 hsa-miR-520a-3p is associated with ferroptosis-related programs in LUSC in connection with MYO19

**Figure 6:**
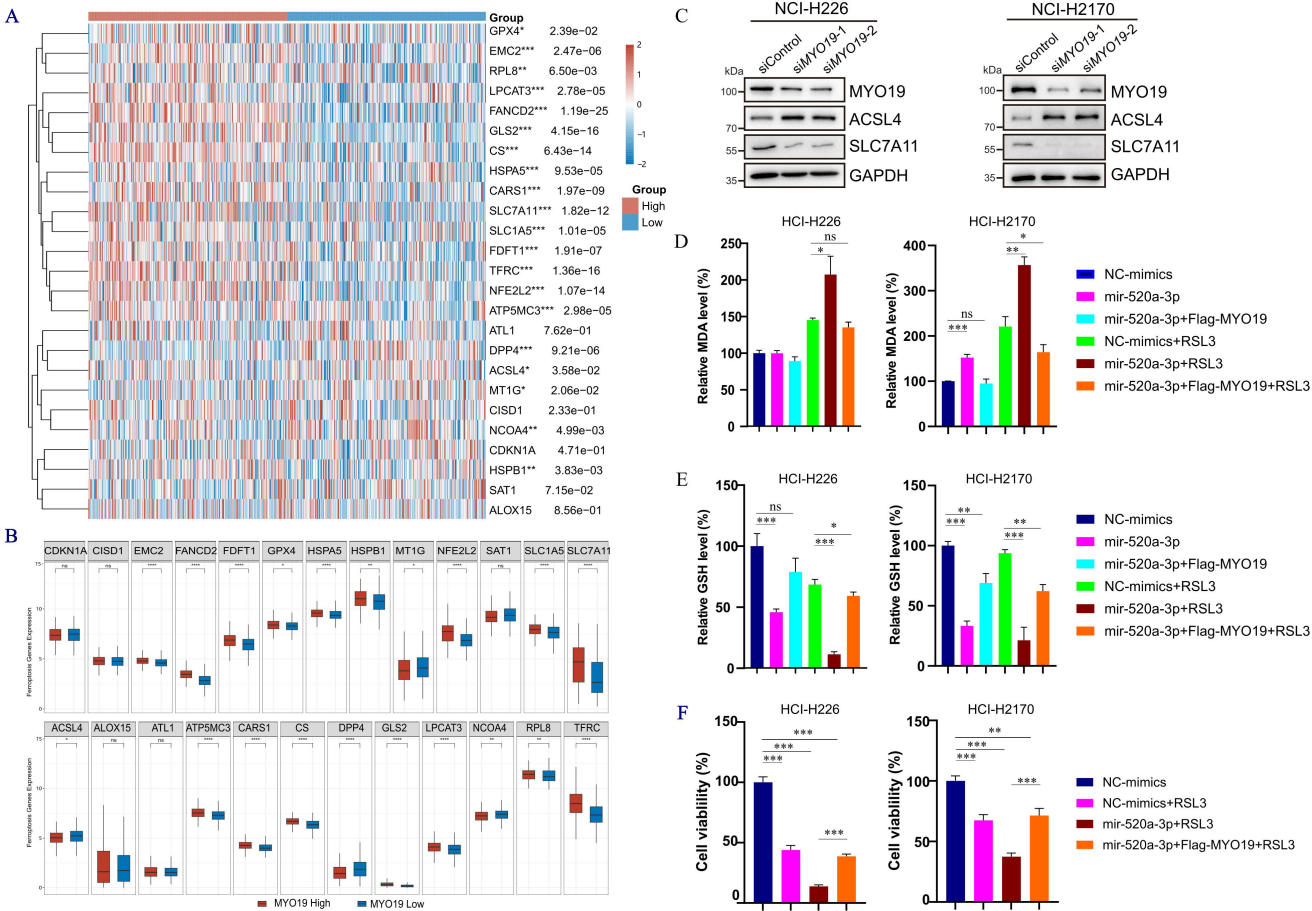
hsa-miR-520a-3p regulates ferroptosis in LUSC by targeting MYO19 and ferroptosis-related genes. **(A)** Heatmap showing the differential expression of ferroptosis-related genes between MYO19-high and MYO19-low groups in LUSC. **(B)** Boxplots comparing the expression levels of key ferroptosis-related genes in MYO19-high and MYO19-low groups. **(C)** The protein expression of MYO19, ACSL4, SLC7A11 were measured in control or MYO19-knockdown NCI-H226 and NCI-H2170 cells. **(D, E)** Quantification of malondialdehyde (MDA) levels and glutathione (GSH) levels in NCI-H226 and NCI-H2170 cells transfected with hsa-miR-520a-3p mimics. **(F)** Cell viability assay in NCI-H226 and NCI-H2170 cells showing the effect of RSL3 (a GPX4 inhibitor) on hsa-miR-520a-3p-overexpressing cells. Bars indicate mean ± SD from ≥3 independent experiments; two-tailed tests with multiple-comparison adjustment where applicable. *p<0.05, **p<0.01, ***p<0.001, ****p<0.0001.

Analysis of ferroptosis-related genes in LUSC revealed distinct expression patterns between the MYO19-high and MYO19-low groups. A heatmap (**Figure 6A**)and Boxplots (**Figure 6B**) show that key ferroptosis inhibitors, including SLC7A11, GPX4, GLS2, HSPA5, FDFT1, and NFE2L2, were upregulated in the MYO19-high group, whereas ferroptosis-promoting genes such as ACSL4 and ALOX15 were downregulated. These *p*-values were not adjusted for multiple comparisons. At the protein level, MYO19 knockdown in NCI-H226 and NCI-H2170 cells resulted in increased ACSL4 and decreased SLC7A11 expression compared with controls (**Figure 6C**). Collectively, these findings suggest that MYO19 suppression may contribute to a ferroptosis-prone cellular state.

To explore cellular correlates, we perturbed hsa-miR-520a-3p in NCI-H226 and NCI-H2170 cells. To investigate the relative contributions of different forms of cell death induced by RSL3, our analysis revealed that the ferroptosis inhibitor Fer-1, rather than the apoptosis inhibitor Z-VAD-FMK, significantly reduced RSL3-induced cell death in NCI-226 cells, suggesting that ferroptosis is the predominant mode of cell death (**Supplementary Figure S4**). Overexpression of hsa-miR-520a-3p significantly decreased GSH (a key ferroptosis safeguard) (**Figure 6E**, p<0.001). Moreover, RSL3 challenge further increased MDA, decreased GSH and reduced cell viability in hsa-miR-520a-3p–overexpressing cells (**Figures 6D–F**, p<0.01), a profile compatible with heightened ferroptosis susceptibility. To determine whether the protein changes associated with miR-520a-3p act through MYO19, a rescue experiment was performed in NCI-H226 cells. The results demonstrated that re-expression of MYO19 in hsa-miR-520a-3p-overexpressing cells reversed all the observed phenotypic alterations (**Figures 6D–F**). These findings suggest that hsa-miR-520a-3p may influence ferroptosis in a MYO19-dependent manner, although further validation is needed to establish the causal relationship.

Collectively, multi-omics analyses and *in vitro* assays suggest a potential link between the hsa-miR-520a-3p/MYO19 axis and ferroptosis-related signatures in LUSC, with MYO19 overexpression potentially correlating with ferroptosis resistance, and hsa-miR-520a-3p expression correlating with increased ferroptosis susceptibility. However, these findings are correlative and require further mechanistic and *in vivo* validation.

## 4 Discussion

Lung cancer remains a major contributor to global cancer mortality, with LUSC exhibiting particular therapeutic challenges (21). Several studies have highlighted the importance of ferroptosis-related genes and immune microenvironment alterations in influencing cancer prognosis and treatment responses. Here, we found MYO19, a mitochondrially localized myosin, was significantly overexpressed in LUSC and associated with poor prognosis, advanced tumor stages and reduced immune infiltration, suggesting that MYO19 has a certain diagnostic and prognosis value of LUSC treatment (22). Besides, Kaplan-Meier survival analysis showed that patients with high hsa-miR-520a-3p expression or low MYO19 expression had significantly better overall survival. Functional assays demonstrated that hsa-miR-520a-3p directly targeted MYO19, suppressing cell proliferation and migration while enhancing ferroptosis sensitivity. Our study identifies that MYO19 as a potential biomarker associated with LUSC and immune and ferroptosis-related features, which may have implications for clinical stratification and treatment strategies.

Functional rescue experiments further validated the regulatory relationship between hsa-miR-520a-3p and MYO19. In NCI-H226 and NCI-H2170 cells, re-expression of MYO19 partially reversed the ferroptosis-related changes caused by miR-520a-3p overexpression, including elevated ACSL4 and reduced SLC7A11 expression. This partial reversal indicates that MYO19 mediates, at least in part, the influence of hsa-miR-520a-3p on ferroptosis sensitivity. Considering that MYO19 regulates mitochondrial dynamics and redox homeostasis, these results suggest that suppression of MYO19 may lower the ferroptosis threshold by disturbing mitochondrial lipid metabolism. Although this rescue experiment strengthens the evidence for the hsa-miR-520a-3p/MYO19/ferroptosis axis, the relationship remains correlative, and further *in-vivo* or immune-interaction studies are required to confirm causal mechanisms linking MYO19 activity with ferroptosis and immune modulation in LUSC.

Evidence indicates that the development of lung cancer is a complex process including interactions between immune cells with tumor cells. MYO19, a mitochondria-associated myosin motor protein, has garnered increasing attention in cancer research. Emerging studies have demonstrated that MYO19-mediated intracellular motility is critical for maintaining mitochondrial dynamics and functional homeostasis (13). Mechanistically, MYO19 potentially modulates ferroptosis susceptibility by influencing mitochondrial oxidative phosphorylation efficiency and iron metabolism (23). Furthermore, MYO19 may coordinate tumor cell migration and epithelial-mesenchymal transition (EMT) via protein-protein interactions, thereby promoting invasive and metastatic phenotypes (24). Our study found that MYO19 overexpression is associate with poor prognosis with immune-suppressive features in LUSC, suggestive of, but not proving, a role in immune evasion. Ferroptosis, a new form of cell death, is involved in various diseases including cancers, including lung cancer (25). MYO19-deficient can impaired mitochondrial function and enhance ROS generation in cancer cells. However, whether MYO19 regulates ferroptosis and further cancer progression remains to be clarified. Our analysis showed that MYO19 is related to many ferroptosis genes, including SLC7A11, GPX4, GLS2, HSPA5, FDFT1, NFE2L2, ACSL4 and ALOX15. Future studies are needed to further uncover how MYO19 regulates ferroptosis in tumor cells through downstream genes.

MiRNAs serve dual roles as tumor suppressors or oncomiRs, with emerging prognostic value in lung cancer chemoresistance (26). Their dysregulation is implicated in NSCLC progression through regulation of proliferation, apoptosis, and metastasis-related pathways (27, 28). Specifically, miR-150 promotes NSCLC growth via SIRT2/JMJD2A activation, while miR-206 suppresses hepatocellular carcinoma by targeting c-MET (27, 29). Emerging evidence has delineated the tumor-suppressive role of hsa-miR-520a-3p in NSCLC. This miRNA is frequently downregulated in NSCLC tissues, and its restoration significantly suppresses malignant phenotypes-including proliferation, migration, and invasion—while inducing apoptosis (30). Mechanistically, hsa-miR-520a-3p orchestrates dual oncogenic pathway suppression through coordinated inhibition of PI3K/AKT/mTOR-driven survival signaling and RRM2-mediated Wnt/β-catenin activation (30, 31). Notably, RRM2 upregulation correlates with enhanced tumor aggressiveness and chemoresistance, phenotypes that are reversible upon hsa-miR-520a-3p overexpression (31). It is interesting that our identification of hsa-miR-520a-3p as a tumor-suppressive regulator of MYO19 highlights the role of microRNAs in controlling ferroptosis and immune pathways in LUSC.

These findings position ferroptosis-immune crosstalk as a therapeutic frontier in LUSC. Our observation that MYO19-high tumors exhibit both ferroptosis resistance and “cold” microenvironments suggests a potential association with clinical ICI resistance patterns. However, this should not be interpreted as a definitive predictive marker for ICI response without further validation through clinical ICI data (32). Our findings that hsa-miR-520a-3p restores ferroptotic sensitivity and immune cell infiltration suggest a potential role in the efficacy of ICIs in MYO19-high LUSC tumors. Additionally, Chung et al. showed that inducing ferroptosis sensitizes squamous cell carcinomas to ICIs, emphasizing the potential of combining ferroptosis inducers with immunotherapy (33). Our demonstration that hsa-miR-520a-3p promotes ferroptosis through MYO19 suppression provides a basis for exploring this combination in LUSC.

## 5 Conclusion

The regulation of MYO19 by hsa-miR-520a-3p highlights a critical axis influencing tumor progression, immune evasion, and ferroptosis in LUSC. The inclusion of rescue assays provides additional functional evidence that the hsa-miR-520a-3p/MYO19 interaction contributes to ferroptosis-associated molecular changes, while reinforcing the potential of this axis as a therapeutic target in LUSC. Targeting the hsa-miR-520a-3p-MYO19 axis offers a promising strategy for overcoming immune evasion and ferroptosis resistance in LUSC. However, further preclinical and clinical studies are warranted to validate this axis as a therapeutic target and to explore its potential in combination therapies.

## 6 Limitations

This study relies on retrospective data from the TCGA database, which limits the ability to draw causal conclusions and warrants further prospective validation. Our findings were based on *in vitro* experiments in cell-lines, and the lack of *in vivo* tumor or immune-competent models restricts the physiological relevance of these results. Finally, the findings need to be validated in additional LUSC cell lines and patient samples to confirm their generalizability.

## Data Availability

The raw data supporting the conclusions of this article will be made available by the authors, without undue reservation.

## Supplementary material

Supplementary Figure 1 The calibration curve of the overall survival nomogram model in the discovery cohort. The diagonal dashed line represents the ideal nomogram, and the blue, red, and orange lines represent the observed 1-year, 3-year, and 5-year nomograms, respectively.

Supplementary Figure 2 Cross-cancer correlation heatmap of MYO19 and immune checkpoint genes.

Supplementary Figure 3 Expression landscape of hsa-miR-520a-3p across TCGA cancers.

Supplementary Figure 4 Ferroptosis inhibitor fer-1 inhibits RSL3 induced cell death. NCI-H226 cells were pretreated with Z-VAD-FMK or Fer-1 for 12 h, then treated with 20 μM RSL3 for 24 h. The viability of indicated cells were measured by CCK-8 assay. The data represent the mean ± SD from three independent experiments. Statistical analysis by paired Student’s t-test.
