# Supplementary figures and images for: MYO19 is associated with tumor progression, immune evasion, ferroptosis-related signatures in lung squamous cell carcinoma
Source: Front Oncol. 2026 Jan 6;15:1727301. doi: 10.3389/fonc.2025.1727301 (PMC12815804; doi:10.3389/fonc.2025.1727301)

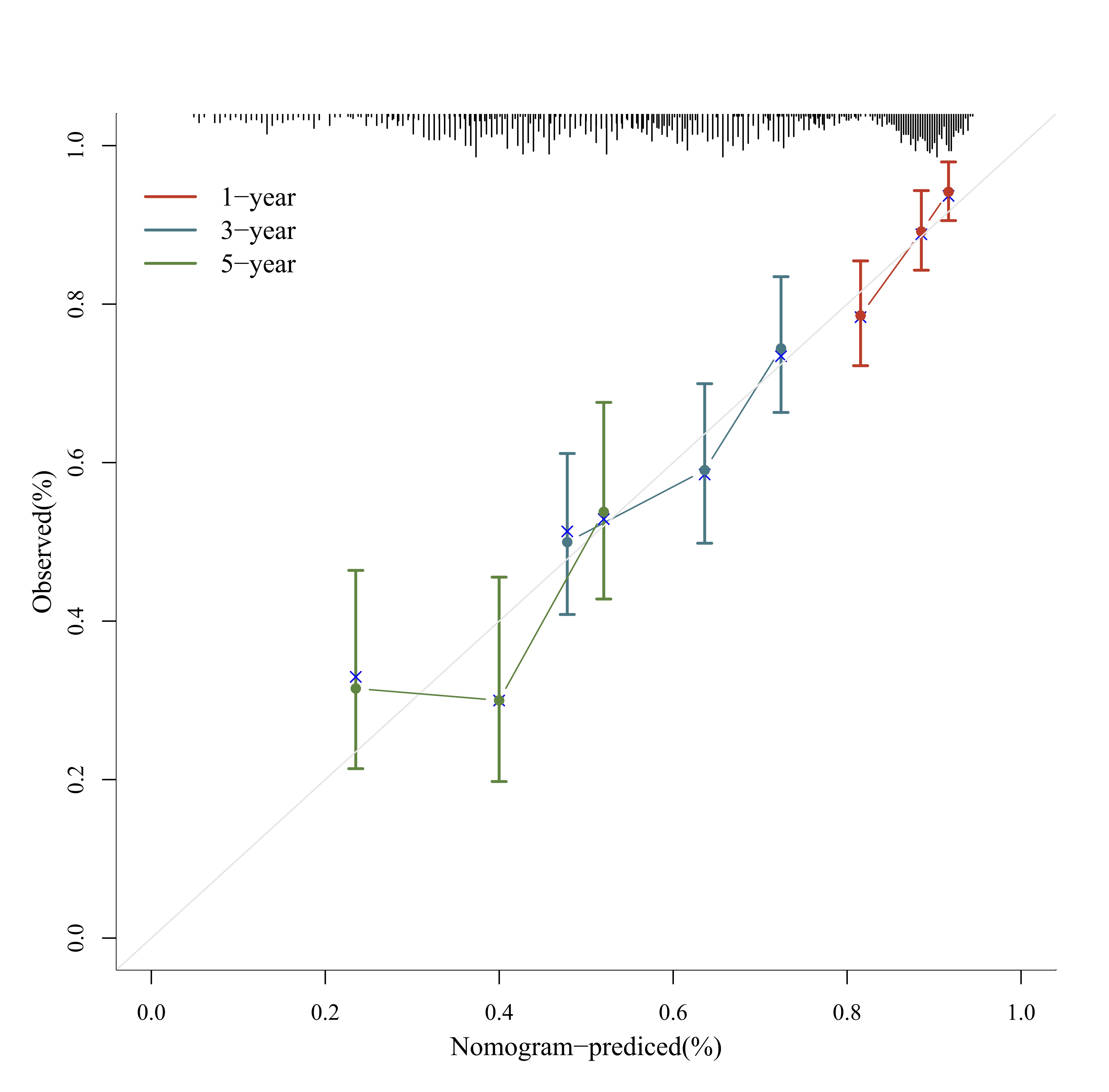

Supplement: Supplementary Figure 1 — The calibration curve of the overall survival nomogram model in the discovery cohort. The diagonal dashed line represents the ideal nomogram, and the blue, red, and orange lines represent the observed 1-year, 3-year, and 5-year nomograms, respectively. [file Image1.jpeg]

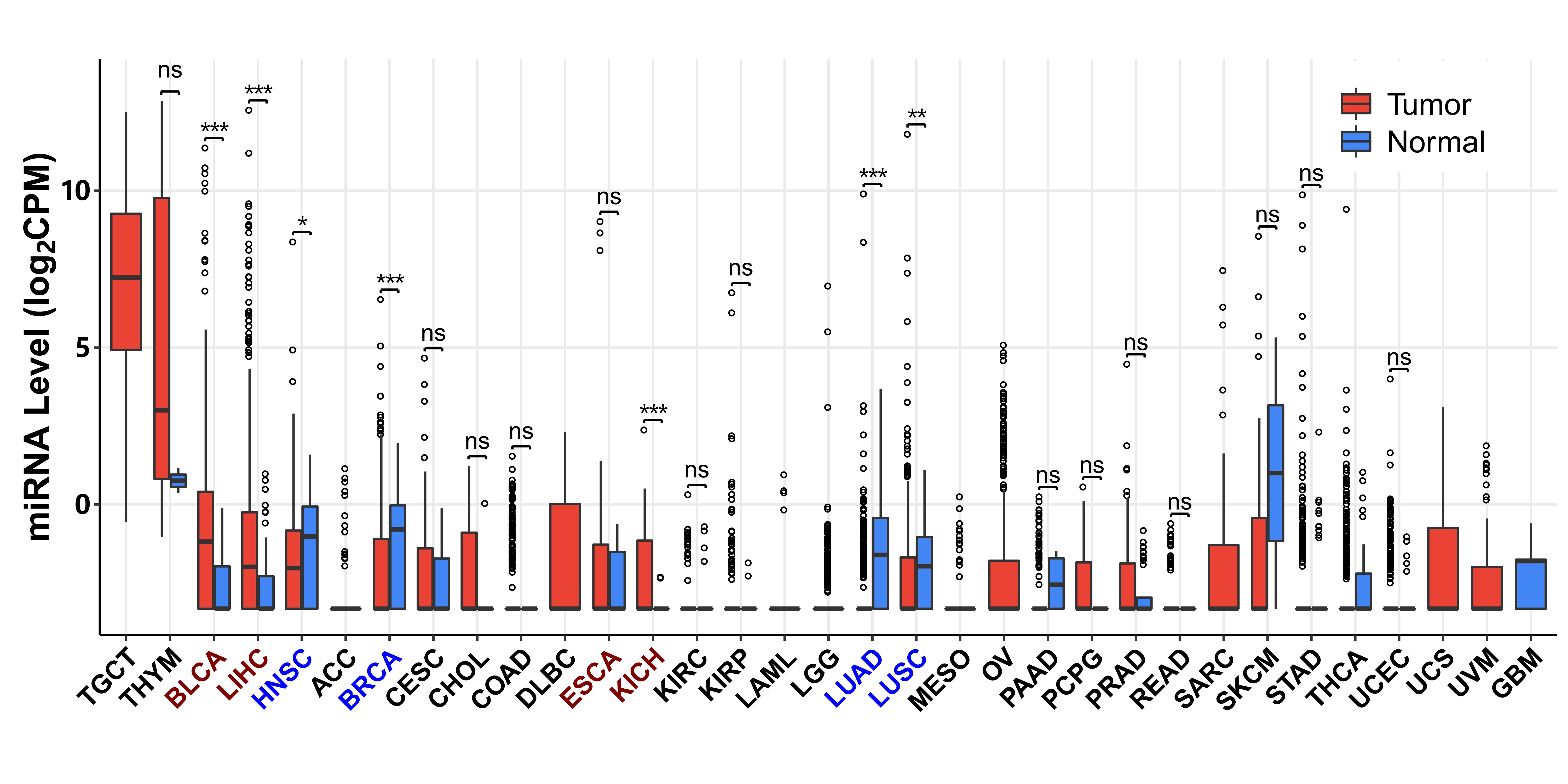

Supplement: Supplementary Figure 2 — Cross-cancer correlation heatmap of MYO19 and immune checkpoint genes. [file Image2.jpeg]

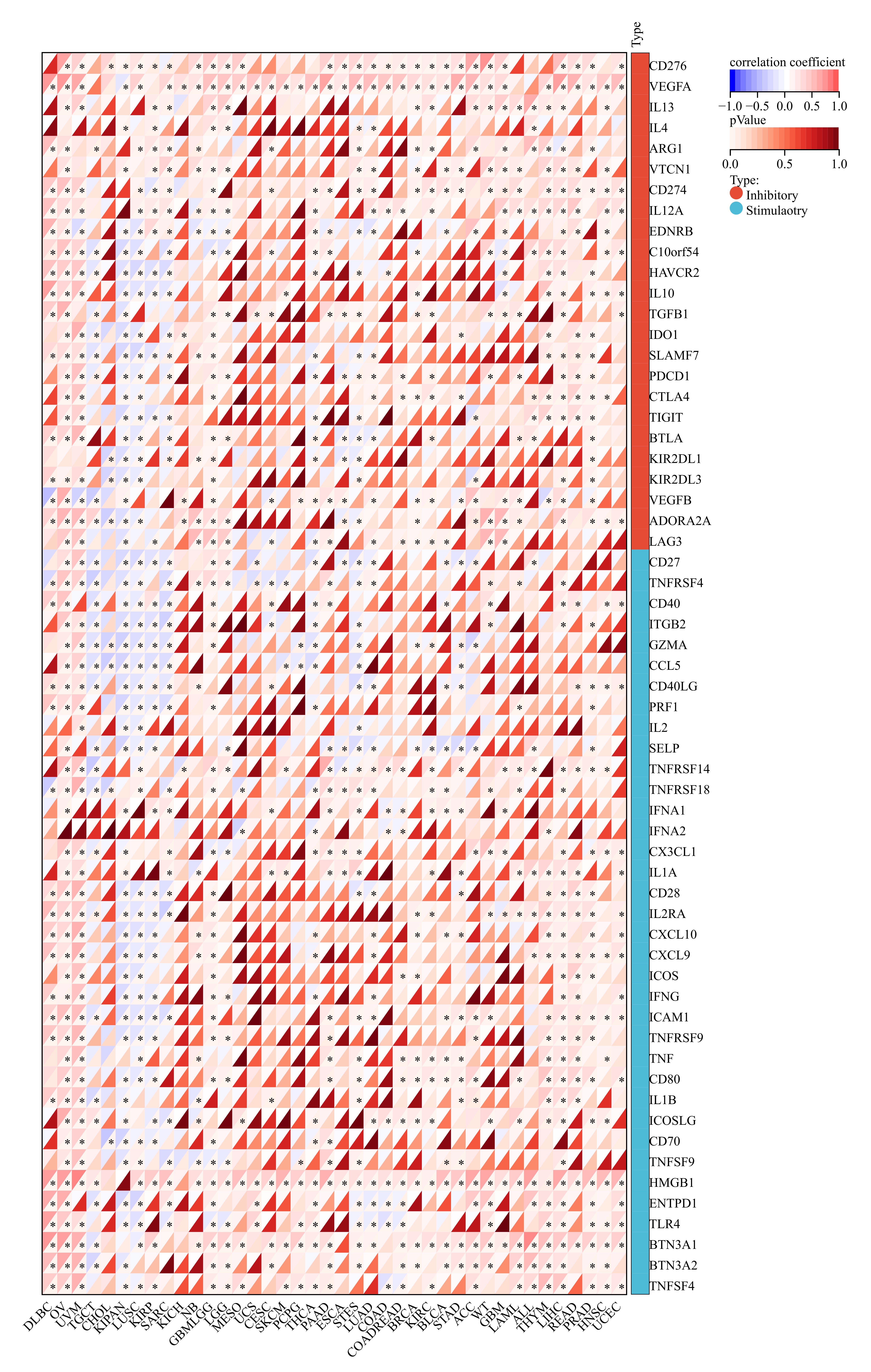

Supplement: Supplementary Figure 3 — Expression landscape of hsa-miR-520a-3p across TCGA cancers. [file Image3.jpeg]

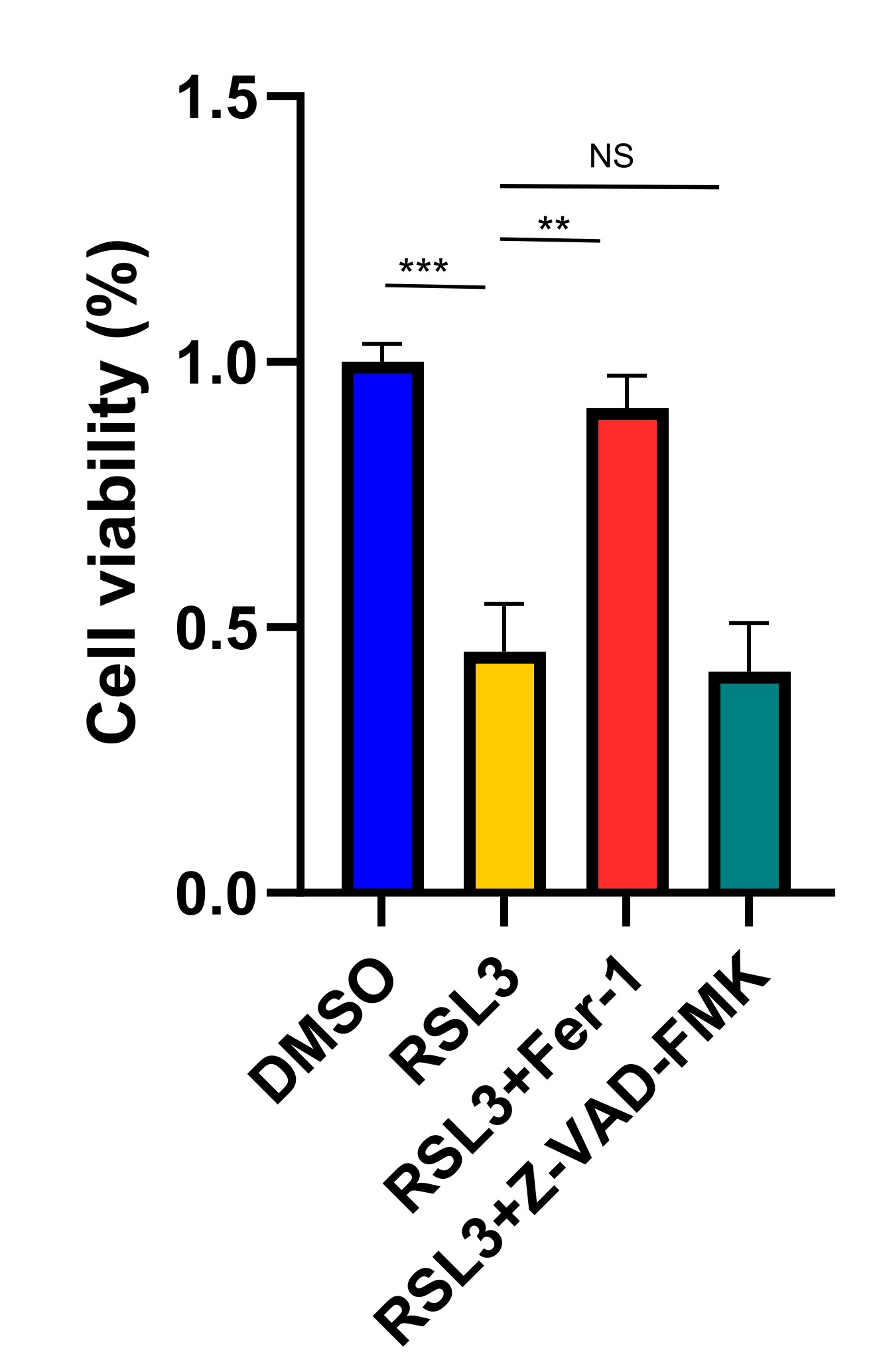

Supplement: Supplementary Figure 4 — Ferroptosis inhibitor fer-1 inhibits RSL3 induced cell death. NCI-H226 cells were pretreated with Z-VAD-FMK or Fer-1 for 12 h, then treated with 20 μM RSL3 for 24 h. The viability of indicated cells were measured by CCK-8 assay. The data represent the mean ± SD from three independent experiments. Statistical analysis by paired Student’s t-test. [file Image4.jpeg]
